# First exploration of radiation temperatures of the laser spot, re-emitting wall and entire hohlraum drive source

**DOI:** 10.1038/s41598-019-41424-6

**Published:** 2019-03-25

**Authors:** Kuan Ren, Shenye Liu, Xufei Xie, Huabing Du, Lifei Hou, Longfei Jing, Dong Yang, Yang Zhao, Ji Yan, Zhiwen Yang, Zhichao Li, Jianjun Dong, Guohong Yang, Sanwei Li, Zhurong Cao, Ke Lan, Wenyi Huo, Jie Liu, Guoli Ren, Yongkun Ding, Shaoen Jiang

**Affiliations:** 1 Research Center of Laser Fusion, China Academy of Engineering Physics, Mianyang, 621900 China; 2 Institute of Applied Physics and Computational Mathematics, Beijing, 100088 China; 3 CAPT, HEDPS, and IFSA Collaborative Innovation Center of MoE, Peking University, Beijing, 100871 China

## Abstract

This study explores the radiation field temperatures introduced by the laser spot, the re-emitting wall in a hohlraum and the entire hohlraum drive source. This investigation, which is the first of its kind, is based on the radiation fluxes from the laser spot and the re-emitting wall, which have been accurately measured using time- and space-resolving flux detectors in a recent work, and additional flux data. The temperature difference between the laser spot and the entire hohlraum drive source was 6.08–35.35% of the temperature of the latter throughout the entire laser pulse, whilst that for the re-emitting wall was 3.90–12.81%. The radiation temperature of the cooler re-emitting wall had more influence on the temperature increase of the entire hohlraum drive source than the hot laser-spot temperature, which has been quantitatively discussed. Experimentally, we established the average distributions of the temperature fields of all the emitting sources, namely laser spot and re-emitting wall, of the irradiating fluxes on the capsule region in the hohlraum radiation field. This important progress in the exploration of radiation temperature distributions within a hohlraum will provide a foundation for determination of the irradiating radiation on the capsule and evaluation of capsule symmetry.

## Introduction

The exploration of the radiation temperature distribution in a hohlraum is of utmost importance in indirectly driven inertial confinement fusion (ICF)^1,2^ because it facilitates the determination of the irradiating radiation on the capsule and evaluation of capsule symmetry^3,4^. For example, in the National Ignition Facility (NIF)^5^, the symmetry of the time-varying capsule drive is controlled by applying two laser rings in each side hohlraum and changing the power ratio of the two rings. This ratio is decided by the time-dependent radiation distribution in the hohlraum.

The hohlraum radiation temperature is usually inferred from the X-ray flux measured by a flat-response X-ray detector (F-XRD)^6,7^ or the DANTE X-ray diagnostic instrument^8,9^ through the laser entrance hole (LEH). However, the recording plane used in the diagnostics is generally of a millimetre order and larger than the LEH, which has a common diameter of approximately 800 *μ*m. When laser beams irradiate the hohlraum, the X-rays emitted by the laser spot, re-emitting wall and capsule are all recorded by the diagnostics. Even though the ViewFactor experiments^10^ at NIF facilitate a direct diagnosis of the X-ray drive from the capsule point of view, because of the challenge outlined earlier, realizing accurate space-resolving flux detection inside the hohlraum in these experiments is still difficult. The creation of additional diagnostic holes on the hohlraum wall has been attempted to measure the laser spot and re-emitting wall fluxes^11^. Nevertheless, the hole closure effect^10,12^ and the change in the hohlraum structure caused by the opening of diagnostic holes could seriously affect the flux results. In addition, even though X-ray framing and streak cameras^13–16^ can provide time- and space-resolving X-ray detection in ICF, quantitatively measuring the X-ray flux using these techniques is difficult. Thus, determining the radiation temperature of the laser spot and re-emitting wall in the hohlraum is also difficult.

In this work, we infer the laser-spot and re-emitting wall radiation temperatures based on the fluxes emitted from those components coupled with additional flux and radiation temperature data obtained as routinised monitoring data in the same experiments. Note that the fluxes emitted from the laser spot and the re-emitting wall have recently been accurately measured using a novel detector known as the time- and space-resolving flux detector (SRFD)^17^. First, the radiation field temperatures introduced by the laser spot, the re-emitting wall and the entire hohlraum drive source were explored. The temperature difference between the laser spot and the entire hohlraum drive source was 6.08–35.35% of the temperature of the latter for the entire laser pulse process. The range for the re-emitting wall was 3.90–12.81%. We also quantitatively discussed the influence of the radiation temperature introduced by the re-emitting wall on the temperature increase of the entire hohlraum drive source compared to that of the laser-spot temperature. The average distributions of the temperature fields of all the emitting sources, namely laser spot and re-emitting wall, of the irradiating fluxes on the capsule region were experimentally established in a hohlraum radiation field.

## Principles of the SRFD

**Figure 1:**
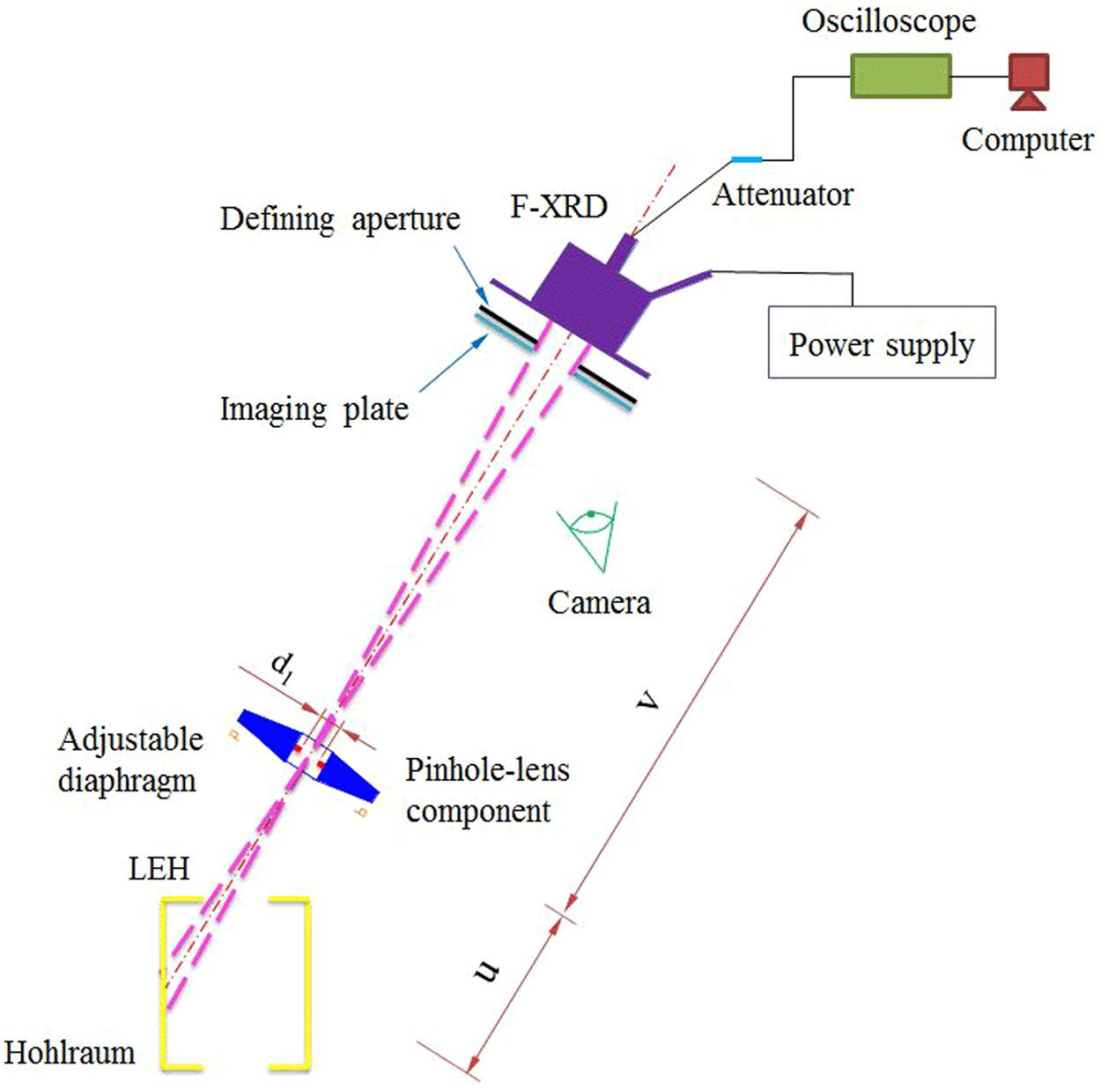
Schematic diagram of the SRFD.

The SRFD is a time- and space-resolving flux detector with a space-resolving capability that differs from the F-XRD^6,7^. Figure 1 shows the SRFD principles. The entirety of each LEH emits X-rays when laser beams irradiate the hohlraum wall through two LEHs. The pinhole in the pinhole lens component is used to create X-ray images of the LEH on the defining aperture, behind which an F-XRD is set. The F-XRD is an absolutely calibrated flux detector for the X-ray energy range of 0.1–4 keV^7^; thus, the fluxes flowing through the defining aperture can be quantitatively detected. In other words, only the X-ray fluxes emitted from the region in the hohlraum LEH corresponding to the defining aperture can be measured. Thus, the spatial resolution can be realised in principle^17^.

An image plate was pasted on the defining aperture with the same area and position hole to record the relationship between the defining aperture and the X-ray image of the LEH (i.e. the relationship between the detection region and LEH). The pinhole was positioned at the centre of an annular lens with the same object distance, image distance and imaging position as the lens. Using visible light to illuminate the hohlraum LEH, the lens can create visible light images of the LEH at the same position as the X-ray images. A camera monitored the relationship between the visible light images and the defining aperture. The lens can assist targeting of the SRFD. In other words, the lens compensated for the disadvantage of pinhole imaging (i.e. the clear aperture of the pinhole was too small to be used for targeting)^17^.

The pinhole diameter was 0.1 mm, and the magnification factor of the pinhole imaging was 10; thus, the spatial resolution was 0.11 mm. The defining aperture diameter was 2 mm; therefore, coupled with the magnification factor, the target and detection area was 0.2 mm, which was larger than the spatial resolution. The X-ray flux detected by the F-XRD was changed to an electric current signal recorded by the oscilloscope. Based on the output voltage signal *V*_(*t*)_, the flux *F*_(*t*)_ can be readily obtained in units of W/cm^2^. For the SRFD employed herein (the schematic diagram of which is shown in Fig. 1), we obtained^17^:

1 $${F}_{(t)}S{\rm{\Delta }}{\rm{\Omega }}/\pi ={F}_{(t)}S{d}_{1}^{2}/4{u}^{2},$$

where *S* is the area of the detection region; ΔΩ is the solid angle of the pinhole relative to the target; *d*_1_ is the pinhole diameter; and *u* is the object distance of the pinhole imaging. The detected X-ray energy is presented as follows^17^:

2 $${F}_{(t)}S{d}_{1}^{2}/4{u}^{2}={V}_{(t)}N/\eta R,$$

where *N* is the signal attenuation factor; *η* is the response sensitivity of the F-XRD; and *R* is the electric resistance of the oscilloscope. Thus, the detected flux was obtained from^17^:

3 $${F}_{(t)}=\frac{4N{V}_{(t)}{u}^{2}}{SR\eta {d}_{1}^{2}}.$$

This work focused on the radiation temperature, which characterises the X-ray radiation field in the hohlraum. The temperature *T*_(*t*)_ detected by the SRFD was obtained from^7^:

4 $${T}_{(t)}={(4\frac{{\sin }\theta }{\pi }{F}_{(t)}S/\sigma {\cos }\theta {d}^{2})}^{1/4}={(\frac{16{\tan }\theta N{V}_{(t)}{u}^{2}}{\pi R\eta \sigma {d}_{1}^{2}{d}^{2}})}^{1/4},$$

where *θ* is the SRFD view angle relative to the hohlraum axis; *σ* is the Stefan–Boltzmann constant; and *d* is the detection area diameter. This equivalent method for extracting the laser-spot and re-emitting wall radiation temperatures is considered reasonable, especially from the perspective of energy equivalence in hohlraum surroundings. The relative uncertainties of the temperatures given by SRFD nos 1 and 2 are 3.46% and 3.17%, respectively. The relative uncertainty for the F-XRD was less than 1%^7^.

## Experiments and Experimental Results

**Figure 2:**
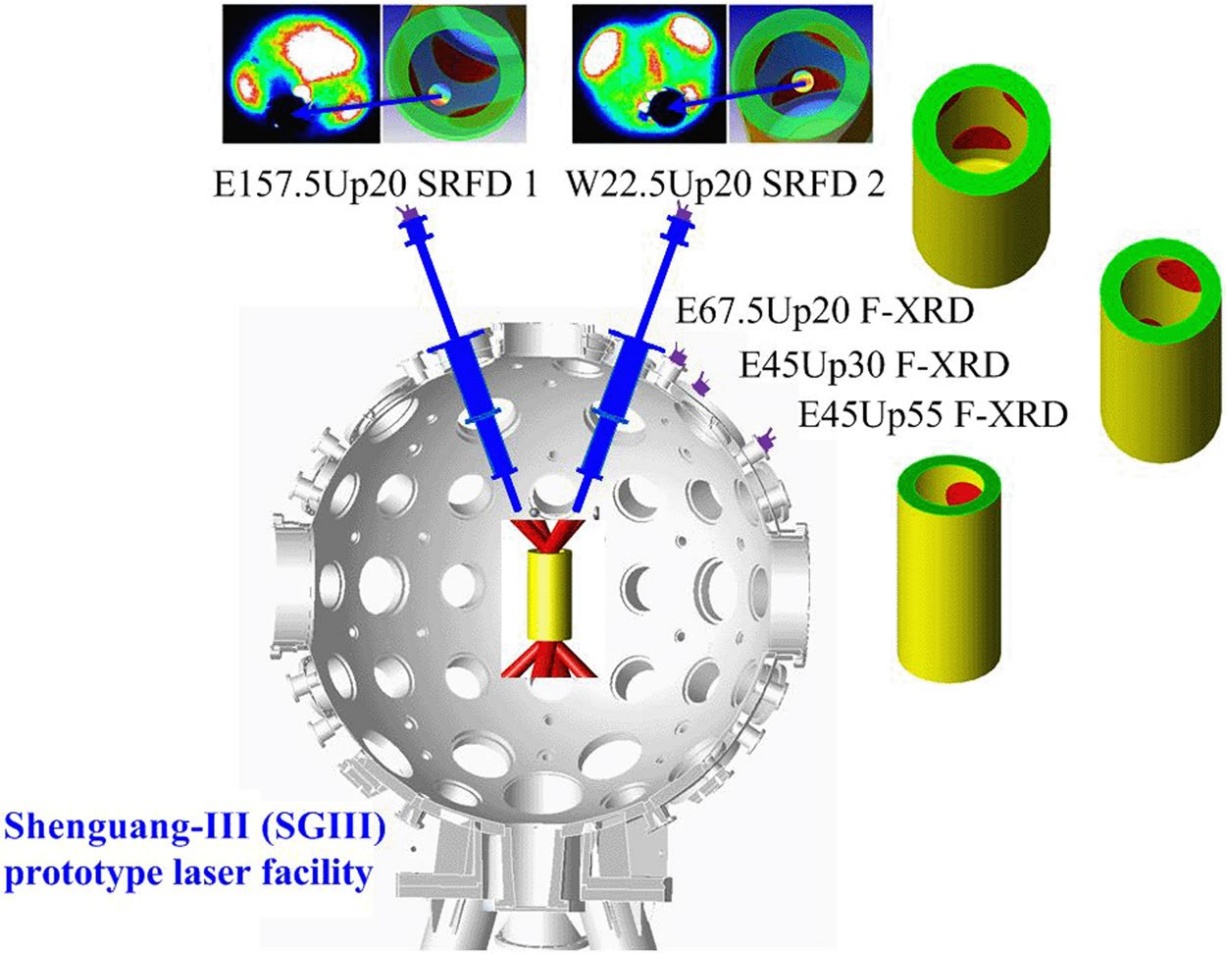
Experimental setup along with the detection regions of SRFD nos 1 and 2 as simulated using the view-factor code (right) and recorded by the image plates (left) and that of the three F-XRDs also simulated using the view-factor code.

The experiments were conducted at the Shenguang-III (SGIII) prototype laser facility^18^. A 1.2-mm-diameter, 2.4-mm-long and 30-*μ*m-thick gold vacuum hohlraum was used to detect the laser spot and re-emitting wall fluxes. This relatively large structure was used to avoid plasma filling and jetting. Figure 2 shows the experimental setup. Eight laser beams (351 nm, 800 J, 3 *ω*) smoothed by the continuous phase plate (CPP) technique, with their 0.5-mm-diameter focal spots, irradiated the hohlraum wall at 45° relative to the hohlraum axis from two 0.85-mm-diameter LEHs positioned symmetrically. The laser beam had a 1.5 ns flat top with 100 ps rising and falling edges. SRFD nos 1 and 2 were positioned at 20° (E157.5Up20 and W22.5Up20) relative to the hohlraum axis with 180° separation in the azimuthal direction and applied to measure the re-emitting wall and laser spot fluxes, respectively. An F-XRD was set at 55° (E45Up55) relative to the hohlraum axis observing the entire LEH flux^17^. Two F-XRDs were set at 20° (E67.5Up20) and 30° (E45Up30) relative to the hohlraum axis to routinely monitor the radiation flux and the temperature from the LEH.

**Figure 3:**
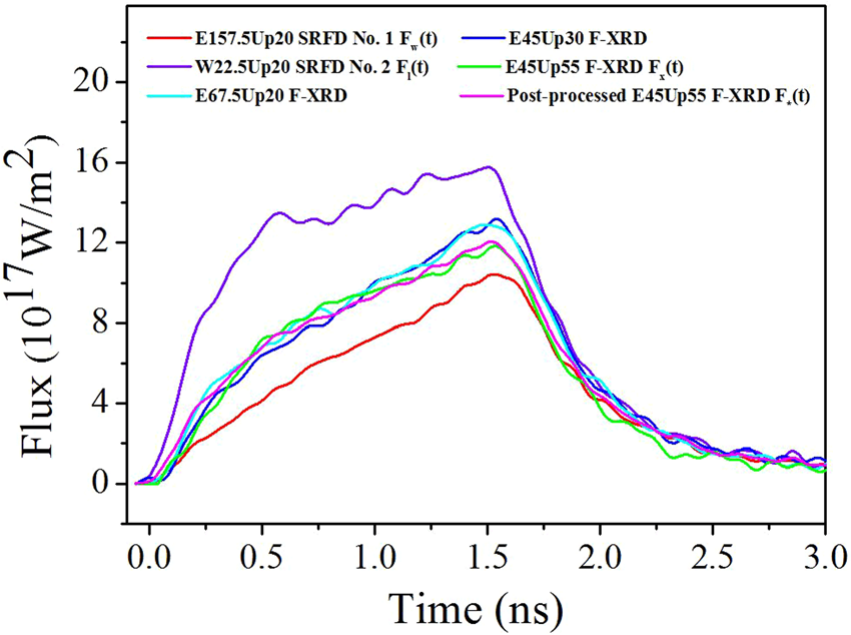
Fluxes detected by SRFD nos 1 (*F*_*w*_(*t*)) and 2 (*F*_*l*_(*t*)) and F-XRDs. The F-XRDs were set at 20°, 30° and 55° (*F*_*x*_(*t*)) relative to the hohlraum axis. The post-processed area-weighted flux (F_*_(*t*)) is also shown.

Figure 2 also shows the detection regions of SRFD nos 1 and 2 as simulated using the view-factor code and recorded by the image plates and that of the three F-XRDs as simulated using the view-factor code. SRFD No. 1 observed the re-emitting wall area, whilst SRFD No. 2 observed the laser spot area. The F-XRDs viewed the LEH, including parts of the re-emitting wall and the laser spots from different angles. Figure 3 depicts the flux results. The fluxes measured by SRFD nos 1 and 2 and the F-XRD set at 55° relative to the hohlraum axis are denoted by *F*_*w*_(*t*), *F*_*l*_(*t*) and *F*_*x*_(*t*), respectively. The areas of the re-emitting wall part and the laser spot parts in the view field of the 55° F-XRD are denoted as *A*_*w*_ and *A*_*l*_, respectively. The post-processed area-weighted flux F_*_(*t*) is then given by^17^:

5 $${F}_{\ast }(t)=\frac{{A}_{l}{F}_{l}(t)+{A}_{w}{F}_{w}(t)}{{A}_{l}+{A}_{w}}.$$

The characteristics of the fluxes have been analysed and explained in a previous work by Ren *et al*.^17^. In this approach, the accuracy was mainly validated by the fact that F_*_(*t*) agreed well with *F*_*x*_(*t*): the disagreement between the peaks of F_*_(*t*) and *F*_*x*_(*t*) was only 1.9%^17^ (Fig. 3).

**Figure 4:**
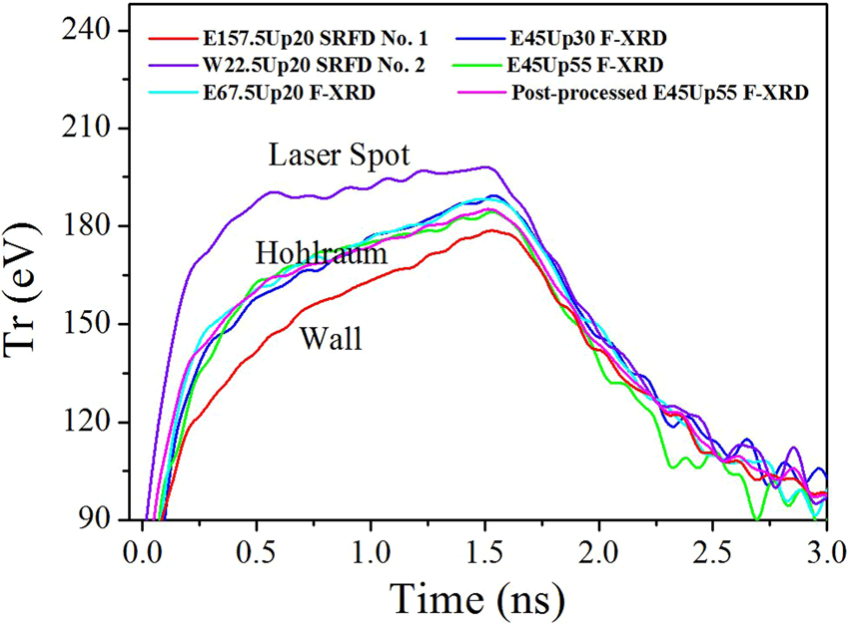
Radiation field temperatures of the laser spot and the re-emitting wall measured by SRFD nos 2 and 1 and the radiation field temperatures detected from the LEH by three F-XRDs set at 20°, 30° and 55° relative to the hohlraum axis. The radiation field temperature obtained from the post-processed area-weighted flux is also shown.

Figure 4 shows the radiation field temperatures of the laser spot and the re-emitting wall measured by SRFD nos 2 and 1, respectively, as determined by referring to Eq. (4) along with the radiation field temperatures detected by the three F-XRDs set at 20°, 30° and 55° relative to the hohlraum axis. The radiation field temperature obtained from the post-processed area-weighted flux referring to Eq. (4) is also shown. Thus, this work can contribute to research geared towards the determination of the radiation field temperatures introduced by the laser spot, the re-emitting wall in a hohlraum and the entire hohlraum drive source.

## Discussion of the Temperature Results

In Fig. 4, the radiation field temperatures measured by the three F-XRDs agreed well, indicating from different angles of observation that the entire hohlraum radiation field is uniform^1^. The largest difference appeared at the peak values of the three temperatures, and was 1.62% of the average peak value. Note that the average value of the three temperatures was used to characterise the entire hohlraum radiation field temperature. The radiation temperature essentially characterises the soft X-rays^19^ and represents the mean kinetic energy of the plasma radiation field in the hohlraum. Moreover, this was one of the most important indices affecting the ignition target design and the success or failure of the ignition effort.

**Figure 5:**
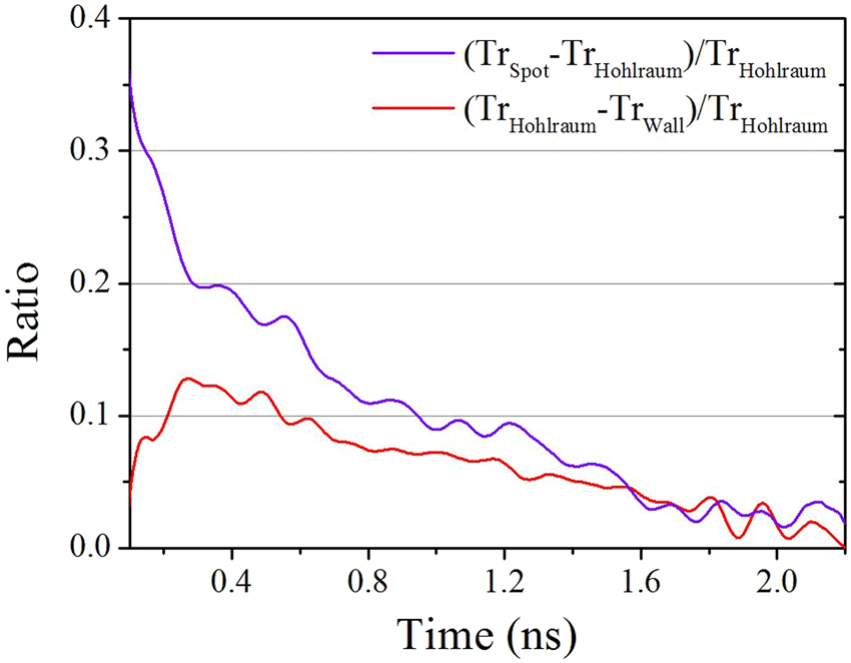
Ratio of the difference between the radiation field temperature introduced by the laser spot and that of the entire hohlraum drive source to the latter ($(T{r}_{Spot}\,-\,T{r}_{Hohlraum})/T{r}_{Hohlraum}$, purple curve) and ratio of the difference between the radiation field temperature of the entire hohlraum drive source and that introduced by the re-emitting wall to the former ($(T{r}_{Hohlraum}\,-\,T{r}_{Wall})/T{r}_{Hohlraum}$, red curve).

Figure 4 also shows that the radiation field temperature of the laser spot rose more quickly than that of the re-emitting wall and to a greater degree, reaching an obvious plateau corresponding to the laser pulse plateau before degrading. The radiation field temperature of the re-emitting wall rose more slowly and directly to a lower peak value. The value of the entire hohlraum radiation field temperature was between that of the other two temperatures. To quantitatively investigate the differences between the radiation field temperature distributions of the laser spot, re-emitting wall and entire hohlraum drive source, Fig. 5 depicts the ratio of the difference between the radiation field temperature introduced by the laser spot and that of the entire hohlraum drive source to the latter ($(T{r}_{Spot}\,-\,T{r}_{Hohlraum})/T{r}_{Hohlraum}$) (purple curve) and the ratio of the difference between the radiation field temperature of the entire hohlraum drive source and that introduced by the re-emitting wall to the former ($(T{r}_{Hohlraum}\,-\,T{r}_{Wall})/T{r}_{Hohlraum}$) (red curve).

**Figure 6:**
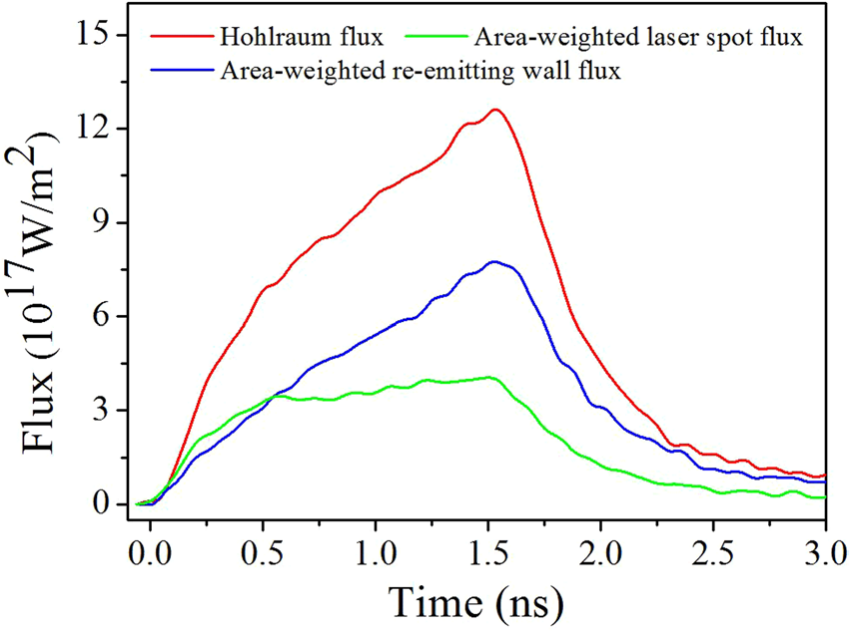
Hohlraum flux (${F}_{Hohlraum}(t)$, red curve), area-weighted laser spot flux (*A*_*l*_*avg*_*F*_*l*_(*t*)/(*A*_*l*_*avg*_ + *A*_*w*_*avg*_), green curve) and area-weighted re-emitting wall flux (*A*_*w*_*avg*_*F*_*w*_(*t*)/(*A*_*l*_*avg*_ + *A*_*w*_*avg*_), blue curve).

Note that the curves were cut before 0.1 ns because the influence of the noise on the temperature values was relatively large before that time; thus, an effective analysis could not be conducted. The purple curve fell directly from 35.35% at 0.1 ns to 6.08% at 1.5 ns. This time behaviour indicated the leading property of the temperature increase of the laser-spot radiation field throughout the entire laser pulse, which was logical. Interestingly, the red curve primarily rose from 3.90% at 0.1 ns to 12.81% at 0.275 ns before falling to 4.55% at 1.5 ns. The rise behaviour can be understood as follows: in the initial 0.1 to 0.275 ns period of the laser drive process, the X-rays emitted by the laser spot irradiated and ablated^3,20^ the cooler re-emitting wall, which absorbed considerable energy^3,20^. The re-emitting wall re-emitted X-rays and established the radiation field. The ablation and absorption processes consumed some of this time; thus, the radiation field temperature of the re-emitting wall relatively slowly rose. However, the rise behaviour of the radiation field temperature of the entire hohlraum was mainly dominated by the laser-spot radiation field temperature, which rose most quickly. Therefore, the red curve rose before the 0.275 ns mark, unlike the falling purple curve. After 0.275 ns elapsed, the radiation field temperature of the re-emitting wall rose quickly after the X-ray ablation and a certain energy absorption, then gradually approached the radiation field temperature of the entire hohlraum drive source. The purple curve was higher than the red curve for the entire laser pulse and fell more rapidly than the red curve after 0.275 ns. This behaviour indicated that the radiation temperature of the cooler re-emitting wall had more influence on the temperature increase of the entire hohlraum drive source than the hot laser-spot temperature. The radiation temperature is quantitatively and closely related to the flux as shown in Eq. (4). We define the average flux of the three fluxes detected by the 20°, 30° and 55° F-XRDs as the hohlraum flux shown in Fig. 6, which is corresponding to the entire hohlraum radiation field temperature characterised in the front with the uniformity. The hohlraum flux is approximately

6 $${F}_{Hohlraum}(t)=\tfrac{{A}_{l\_avg}{F}_{l}(t)+{A}_{w\_avg}{F}_{w}(t)}{{A}_{l\_avg}+{A}_{w\_avg}}=\tfrac{{A}_{l\_avg}{F}_{l}(t)}{{A}_{l\_avg}+{A}_{w\_avg}}+\tfrac{{A}_{w\_avg}{F}_{w}(t)}{{A}_{l\_avg}+{A}_{w\_avg}},$$

where *A*_*l*_*avg*_ is the average area of the laser spot parts in the view fields of the three F-XRDs, and *A*_*w*_*avg*_ is the average area of the re-emitting wall part in the view fields of the three F-XRDs. *A*_*l*_*avg*_*F*_*l*_(*t*)/(*A*_*l*_*avg*_ + *A*_*w*_*avg*_) is the area-weighted laser spot flux, and *A*_*w*_*avg*_*F*_*w*_(*t*)/(*A*_*l*_*avg*_ + *A*_*w*_*avg*_) is the area-weighted re-emitting wall flux, as shown in Fig. 6. The front one is quantitatively smaller than the latter. In addition, the quantitative 0.275 ns and temperature distributions in that period, as shown in Figs 4 and 5, were obtained, which was important for researching the time-varying wall albedo during the foot of an indirect-drive temporally shaped ignition laser pulse. In that foot stage, accurate knowledge of the albedo is most critical for determining the capsule drive symmetry^19^. Neither of the two curves reached zero at the end of the laser pulse, even at 2.2 ns in Fig. 5, which implies that the non-uniform radiation field temperature of the hohlraum was maintained throughout the entirety of the laser pulse in a space-resolving sense.

In Fig. 4, the disagreement between the peaks of the radiation field temperature detected from the LEH by the F-XRDs set at 55° relative to the hohlraum axis and the radiation temperature obtained from the post-processed area-weighted flux was only 0.47%; thus, the measured time-dependent radiation temperature of the laser spot (Fig. 4) could represent an average value of all the laser spot temperatures. Accordingly, that of the re-emitting wall (Fig. 4) could represent an average value of all the re-emitting wall temperatures. These results indicated that the influence of plasma filling and jetting on the radiation temperatures could indeed be avoided using a relatively large hohlraum structure coupled with the detection regions shown in Fig. 2. As a result, the average distributions of the temperature fields of all the emitting source, namely laser spot and re-emitting wall, of the irradiating fluxes on the capsule region could be established in a hohlraum, experimentally. This precise exploration of the time-dependent radiation temperature distributions in a hohlraum will promote an optimal design of the ignition hohlraum structure and further the advancement of the fusion ignition quality and drive symmetry of the capsule. Thus, we believe this work has extreme significance and practical value for the design of the ignition target and the promotion of the development of hohlraum energetics.

## Conclusions

In summary, we firstly determined the radiation temperatures of the laser spot, re-emitting wall and entire hohlraum for a hohlraum under ICF using previous flux measurements^17^ and additional flux data routinely monitored from the LEHs. The temperature difference between the laser spot and the entire hohlraum drive source was 6.08% to 35.35% of the temperature of the latter for the entire laser pulse with a direct degradation process. The difference for the re-emitting wall was 3.90% to 12.81% with an increasing and decreasing process explained by the X-ray ablation and certain time-consuming energy absorption processes. In addition, the radiation temperature of the cooler re-emitting wall had more influence on the temperature increase of the entire hohlraum drive source compared to the hot laser-spot temperature, which is quantitatively discussed. Experimentally, we established the average distributions of the temperature fields of all the emitting sources, namely laser spot and re-emitting wall, of the irradiating fluxes on the capsule region in the hohlraum.

This important advancement in the quantitative exploration of the time-dependent temperature distributions within a hohlraum will be essential for obtaining the irradiating radiation on the capsule and evaluation of capsule symmetry. Moreover, this is a significant and innovative progress in the research on capsule-driven properties. Further work in incorporating the view-factor code is ongoing. However, several issues still remain to be resolved. For example, in this work, we only used the vacuum hohlraum for basic and necessary explorations; evaluating the influence of the capsule on the hohlraum radiation field distributions is still a problem. Furthermore, the scale of the capsule in the implosion process is time-varying; a method for determining this scale is also worth researching.
